# Predictors of life satisfaction in fathers of children with cerebral palsy

**DOI:** 10.1590/1984-0462/2025/43/2025001

**Published:** 2025-11-14

**Authors:** Atahan Turhan, Menşure Aslan, Başak Çiğdem Karaçay

**Affiliations:** a Kırşehir Ahi Evran University, Department of Physical Therapy and Rehabilitation, Kırşehir, Turkey.

**Keywords:** Caregivers, Cerebral palsy, Life satisfaction, Motor function, Cuidadores, Paralisia cerebral, Satisfação com a vida, Função motora

## Abstract

**Objective:**

The aim of this study was to examine the determinants of life satisfaction among fathers of children diagnosed with cerebral palsy (CP).

**Methods:**

A total of 122 children diagnosed with CP and their fathers were included in the study. Sociodemographic data and clinical characteristics of the children with CP and their fathers were recorded in the Personal Information Form. The life satisfaction level of the fathers was assessed using the Satisfaction with Life Scale (SWLS). The Gross Motor Function Classification System was used to assess the motor function in children with CP.

**Results:**

The results of the study showed that fathers’ SWLS score was significantly correlated with time to diagnosis of child (TDC) with CP (r=-0.394), the number of siblings (NSC) (r=-0.213), and children’s motor function level (r=-0.270) (p<0.05). According to the results of multiple regression analysis, children’s motor function level, NSC, and TDC were found to be independent and significant predictors of SWLS, explaining 20% of the variance (p<0.05).

**Conclusions:**

Fathers’ life satisfaction was significantly affected in families with children with CP. In addition, children’s motor function level, NSC, and TDC were found to be determinants of fathers’ life satisfaction.

## RESUMO

**Objetivo:**

O objetivo deste estudo é examinar os determinantes da satisfação com a vida entre os pais de crianças com diagnóstico de Paralisia Cerebral (PC).

**Métodos:**

Foram incluídas 122 crianças com diagnóstico de PC e os seus pais. Os dados sociodemográficos e as características clínicas das crianças com PC e de seus pais foram registrados no Formulário de Informações Pessoais. O nível de satisfação com a vida dos pais foi avaliado através da Escala de Satisfação com a Vida (ESV). O Sistema de Classificação da Função Motora Grossa foi utilizado para avaliar a função motora em crianças com PC.

**Resultados:**

Os resultados do estudo mostraram que a pontuação ESV dos pais estava significativamente correlacionada com o tempo até o diagnóstico da criança (TD) com PC (r=-0.394), o número de irmãos (r=-0.213) e o nível de função motora da criança (r=-0.270) (p<0.05). De acordo com os resultados da análise de regressão múltipla, o nível de função motora da criança, o número de irmãos e o TD foram considerados preditores independentes e significativos da ESV, explicando 20% da variância (p < 0.05).

**Conclusões:**

A satisfação com a vida dos pais foi significativamente afetada nas famílias com crianças com PC. Além disso, o nível de função motora das crianças, o número de irmãos e o TD foram considerados determinantes da satisfação com a vida dos pais.

## INTRODUCTION

 Cerebral palsy (CP) is defined as "a group of permanent disorders of the development of movement and posture, causing activity limitation, that are attributed to non-progressive disturbances that occurred in the developing fetal or infant brain. The motor disorders of cerebral palsy are often accompanied by disturbances of sensation, perception, cognition, communication, and behavior, by epilepsy, and by secondary musculoskeletal problems".^1^ Recently, the prevalence of CP was reported to be between 2 and 2.5 per 1000 births.^2^ Current data do not indicate a clear global decline in the prevalence of CP. However, while a decrease in CP prevalence has been reported in high-income countries, it has been stable or increasing in low- and middle-income countries.^3^ Additionally, the increase in important risk factors for CP, such as prematurity and low birth weight, raises concerns that this may increase the prevalence of CP in the future, especially in low- and middle-income countries.^4^ Having a child diagnosed with CP is a difficult process for parents and other family members to adapt to. Additionally, problems such as not having enough information about the disease and not being able to predict the course of the disease negatively affect parents’ health, quality of life, as well as social, physical, and emotional well-being.^5^

 Life satisfaction is a general expression of one’s feelings and attitudes about life, reflecting negative or positive changes over a certain period. It is one of the most important indicators of an individual’s mood during certain life periods. Life satisfaction varies with the emotional reactions such as happiness and sadness that the person exhibits during life events.^6-8^ Among the factors affecting life satisfaction are sociodemographic characteristics such as age, sex, occupation, and financial status.^9,10^ Parents of children with CP have anxiety disorders, depressive mood, and low levels of life satisfaction.^11^ Although the emotional and caregiving burdens on mothers have been frequently documented in the literature, the experiences of fathers remain largely underexplored.^12^ However, fathers also play a vital role in the caregiving process, and their psychological well-being significantly influences family functioning and the quality of care provided to the child.^13^

 Improving the life satisfaction of fathers of children with CP is essential not only for their personal well-being but also for the overall development and care of their children.^14^ This study aims to address the gap in the literature by identifying the factors that affect life satisfaction among fathers and by exploring how these factors impact their emotional health and family dynamics. 

## METHOD

 This cross-sectional study was approved by the Clinical Research Ethics Committee of Kırşehir Ahi Evran University Faculty of Medicine (Decision number 2023/121, Dated 06/06/2023) and was undertaken in accordance with the principles of the Declaration of Helsinki. This study was also registered in the Registry of Clinical Trials (Registration Number: NCT06437340). The registration can be accessed at: https://clinicaltrials.gov/study/NCT06437340. The study was conducted at Kırşehir Ahi Evran University Physical Therapy and Rehabilitation Center between July 2023 and May 2024. Participants were selected from among fathers with children diagnosed with CP who were receiving physiotherapy at these centers and who met the inclusion–exclusion criteria. The research data were collected through face-to-face interviews by researchers at the center. All participants were given comprehensive information about the purpose of the study and signed the informed consent forms. 

**Figure 1:**
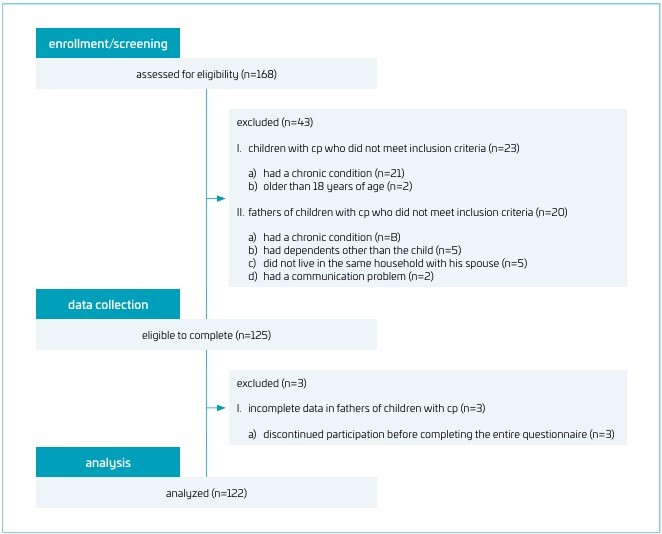
The study’s flowchart.

 The study was conducted with 122 fathers of children with CP. Inclusion criteria were that fathers had the ability to read and write without communication difficulties, had a biological child with CP aged between 1 and 18 years, and the child had no other medical diagnosis, and the children was receiving regular physiotherapy and rehabilitation support. Additionally, they needed to live in the same house with their spouse and child or children and be willing to participate in the study. Fathers were excluded if they had dependents other than the child with CP (e.g., disabled, elderly, and chronically ill) or if they had a chronic condition that could affect life satisfaction (e.g., neurological, rheumatological, and psychiatric conditions). The study’s flowchart is given in Figure 1. 

 Sociodemographic and clinical information of the participants was recorded using the Personal Information Form designed by the investigators. Life satisfaction of the fathers of children with CP was recorded with the Satisfaction with Life Scale (SWLS), and the motor function level of the children was recorded with the Gross Motor Function Classification System (GMFCS). 

 Sociodemographic and clinical data of the children with CP, including sex, age, height, weight, duration of physical therapy, number of siblings (NSC), birth order, type of disability, support received from outside of the family, number of surgical operations, time to diagnosis of child (TDC), and ambulation status, were recorded. Sociodemographic data of the fathers, such as age, height, weight, period of active employment, education level, occupation, monthly income, and life satisfaction level, were recorded. Additionally, data on the total income of the family and the number of people living at home were collected. 

 SWLS developed by Diener et al. was used to assess the life satisfaction of the participants. The SWLS is a 7-point Likerttype scale consisting of five items. Each item of the scale is scored between 1 and 7, and the total score ranges between 5 and 35. The SWLS is categorized as follows: 31–35 points (≥85th percentile) extremely satisfied; 26–30 points (59th–84th percentile), satisfied; 21–25 points (29th–58th percentile), slightly satisfied; 20 points (20th–28th percentile), neutral; 15–19 points (6th–19th percentile), slightly dissatisfied; 10–14 points (2nd–5th percentile), dissatisfied; and 5–9 points (1st percentile), extremely dissatisfied. A higher total score indicates greater life satisfaction. The test–retest correlation coefficient of this scale was 0.82.^15^ In the current study, the SWLS was used as a raw score. 

 GMFCS was used to examine the gross motor function level of children with CP. The GMFCS classifies the gross motor function levels of children from age 1 to 5. Level 1 is the highest, and Level 5 is the lowest function level. A higher level represents a lower status of functionality.^16-18^

 The sample size calculation was conducted using G*Power 3.1 software, employing the linear multiple regression test as the basis. Previous research by Shivers and Resor^19^ identified child disability status, child health, and paternal education level as significant predictors of fathers’ health status. Furthermore, child health, poverty, educational attainment, and immigrant status were found to be significantly associated with fathers’ life satisfaction. Based on these findings, the minimum required sample size for the present analysis was determined to be 117 participants, assuming an effect size of 0.06, a significance level (alpha) of 0.05, and a statistical power of 80%. 

 IBM’s Statistical Package for Social Sciences (SPSS Inc., Chicago, IL, USA) version 25.0 was used to analyze the collected data. Descriptive statistical information was given as mean and standard deviation (X±SD). Categorical variables were given as percentages. The conformity of the data to the normal distribution was evaluated by visual (histograms and probability plots) and analytical methods (Kolmogorov-Smirnov test). In the analysis of bivariate relationships between variables, the Pearson correlation coefficient was used to examine relationships between quantitative variables; the Eta coefficient (η^2^) was used to evaluate relationships between nominal and metric variables; the Polyserial correlation coefficient was used to estimate relationships between ordinal and metric variables. The correlation coefficient was interpreted as follows: 1.00=perfect, 0.70–1.00=high, 0.70–0.30=moderate, and 0.00–0.30=weak correlation.^20^ Multiple linear regression analysis was used to determine the effect of the participants’ demographic and clinical characteristics on their life satisfaction level. The level of statistical significance was 0.05. 

## RESULTS

 A total of 122 children with CP and their fathers were included in this study (mean age of children: 6.50±4.68 years, mean age of fathers: 41.44±11.15 years). The clinical profiles and sociodemographic features of children with CP and the sociodemographic characteristics of their fathers are summarized in Table 1. 

**Table 1:** Sociodemographic characteristics of children with cerebral palsy and their fathers.

Variables (fathers)		Mean±SD	Min–Max
Age (years)		41.44±11.15	20–64
Height (cm)		177.52±7.19	160–192
Weight (kg)		78.50±8.86	60–100
BMI (kg/m^2^)		25.13±3.72	17.09–33.21
Duration of working life (years)		23.46±12.07	3–37
Monthly income (TL)		21899.20±10187.71	8600–60,000
Total family income (TL)		40450.16±16062.32	8600–95,000
Number of people living at home		4.10±0.95	3–8
Satisfaction with Life Scale		13.90±3.13	5–23
Variables (children)		Mean±SD	Min–Max
Age (years)		6.50±4.68	1–18
Height (cm)		121.15±25.78	70–166
Weight (kg)		29.47±14.01	9–68
Time to diagnosis of child (years)		3.63±1.57	1–7
Number of siblings of the child		2.20±1.60	0–8
Duration of child’s physical therapy (years)		5.09±4.17	1–15
		n	%
Gender			
	Male	64	52.5
	Female	58	47.5
RCAS			
	No siblings	22	18.0
	First	52	42.6
	Second	37	30.3
	Third	9	7.4
	Fourth and more	2	1.6

SD: standard deviation; TL: Turkish Lira; RCAS: ranking of the child among siblings; BMI: body mass index.

 SWLS has a statistically significant moderate negative correlation with TDC (r=-0.394, p=0.000), a weak negative correlation with GMFCS (r=-0.270, p=0.003), and a weak negative correlation with NSC (r=-0.213, p=0.018) (Table 2). 

**Table 2:** Correlation analysis between dependent and independent variables.

Variables	The satisfaction with life scale
Father’s age	0.043*
Father’s height	0.144*
Father’s weight	0.115*
Duration of father’s working life	0.089*
Father’s monthly income	-0.090*
Total family income	-0.128*
Number of people living at home	0.025*
Father’s education level	-0.018†
Father’s occupation	-0.079‡
Age of the child	-0.028*
Child’s height	-0.136*
Child’s weight	-0.015*
Time to diagnosis of child	-0.394*,§
Number of siblings of the child	-0.213*,//
Duration of child’s physical therapy	0.070*
Gender of the child	0.072‡
Ranking of the child among siblings	0.141†
Child’s type of disability	0.060‡
Gross motor function classification system	**-0.270†,§**
Number of surgical operations the child underwent	-0.095‡
Non-family support in child care	-0.117‡

*Pearson’s correlation coefficient; †Polyserial correlation coefficient; ‡Eta correlation coefficient (η2); §p<0.01; //p<0.05.

Statistically significant values are denoted in bold.

 According to the multiple regression analysis results, the predicted TDC, GMFCS, and NSC scores were independent and significant determinants of SWLS and explained 20% of the variance (p<0.05) (Table 3). 

**Table 3:** Determinants of life satisfaction levels of fathers of children with cerebral palsy.

Variables	B	SE	Beta	p-value
Constant	18.587	0.851	-	<0.001*
TDC	-0.652	0.168	-0.328	0.001*
GMFCS	-0.480	0.203	-0.199	0.020*
NSC	-0.408	0.160	-0.208	0.012*

Summary of model: R=0.47; R2=0.22; Adjusted R2=0.20 (p<0.001).

*p<0.05.

TDC: time to diagnosis of child; GMFCS: Gross Motor Function Classification System; NSC: number of siblings of the child; B: unstandardized regression coefficient; SE: standard error.

The regression equation was obtained as follows:18.587+(−0.652×TDC)+ (−0.480×GMFCS)+(−0.408×NSC).

## DISCUSSION

 According to this study’s results, the GMFCS level of children with CP, the NSC, and TDC were independent and significant determinants of fathers’ SWLS level. The increase in the level of disability in children with CP may make it difficult for fathers to care for their children. Additionally, late diagnosis of children may decrease fathers’ life satisfaction levels. Increasing the NSC will increase the number of children requiring care, which will be an additional burden on fathers. These situations will affect the life satisfaction of fathers with children with CP. 

 Few studies have been found to date on parents of children with CP, especially fathers, in the literature. This may be because the mother is usually the primary caregiver. In one study, no difference was found between mothers and fathers of children with CP in terms of experienced stress. However, while mothers experience more parenthood-related stress, fathers experience more child-related stress.^21^ In another study, it was observed that the levels of caregiver burden, anxiety, and depression symptoms of mothers and fathers of CP children were similar.^22^ A review of the literature reveals that most studies focus on mothers of children with CP, with fathers largely excluded from the research. Nevertheless, despite cultural differences, these fathers can be affected by this situation to the same extent as mothers. Fathers are often responsible for providing financial support, facilitating transfers, and mobilizing resources while caring for a child with CP. Therefore, biological fathers who live with their child with CP and their spouse and who have to take active responsibility in the management and care of the child with CP were included in this study. The life satisfaction of a father of a child with CP may vary depending on several factors. Fathers of children with CP tend to feel lower life satisfaction compared to fathers of children with normal development. Wang et al. reported that parents of children with CP have low levels of life satisfaction.^23^ Darling et al. stated that fathers of children with disabilities experience high levels of stress, which negatively affects life satisfaction.^24^ Shivers and Resor reported that parents of children with physical disabilities had lower levels of life satisfaction than parents of healthy children.^19^ Lu et al. stated that parents of children with abnormal development had lower life satisfaction than parents of children with normal development.^25^ According to the study by Johansen et al., parents of children with rare diagnoses had lower levels of life satisfaction.^26^ In accordance with the literature, life satisfaction levels of fathers of children with CP were low in this study. 

 The level of disability of children with CP was significantly related to their parents’ level of life satisfaction. In a study by Esdaile and Greenwood, the severity of disability in children with CP was associated with the level of stress in parents. Their results showed that an increased disability level in children leads to increased stress experienced by parents.^27^ According to the study by Wang and Jong, the stress felt by parents of children with CP was significantly related to the child’s age and motor disability level. It has also been reported that the increasing level of disability in children increases the level of stress in parents.^28^ Millere and Senkane reported that increased stress levels of parents of disabled children negatively affect life satisfaction.^29^ In our study, stress level was not questioned, but gross motor dysfunction was found to be a factor affecting life satisfaction. Gross motor dysfunction is a condition that makes the child’s transfer difficult and also brings more clinical medical problems (hip dislocation, scoliosis, and contractures). These may affect the determination of gross motor dysfunction as a factor affecting the life satisfaction of fathers with children with CP. The study by Aktan et al. stated that parents of individuals with multiple disabilities have a lower life satisfaction levels than parents of individuals with a single disability.^30^ In the current research, the NSC of children with CP and TDC was a significant predictor of their fathers’ life satisfaction. Gómez-Ortiz and Sánchez-Sánchez stated that the life habits of parents changed after the birth of their first child, due to the many problems they faced. They reported that the increase in the number of children accompanied by changes in living habits negatively affected life satisfaction.^31^ In another study, it was reported that parents with low levels of life satisfaction have the view that increasing the number of children they have will further reduce the level of life satisfaction.^32^ In our study, only fathers with a single disabled child were included in the study; however, the NSC of children with CP was also found to be a factor affecting the life satisfaction of fathers. An increase in the number of children in the family causes more responsibility and financial burden for the father. This may have contributed to the finding of the NSC as a factor affecting life satisfaction in our study. 

 Novak et al. reported that delayed diagnosis of children with CP has a negative impact on their development, which in turn leads to parental dissatisfaction and depression.^33^ te Velde et al. stated that early diagnosis in children with CP allows parents to provide psychological support.^34^ In accordance with the literature, the findings of this study show that a longer time since diagnosis and an increase in the NSC negatively affect the life satisfaction of fathers of children with CP. A longer time until diagnosis leads to more frequent doctor appointments and medical examinations compared to a healthy baby developing during the same period, creating both a caregiving and financial burden during this process. In addition, the developmental delay and uncertainty in the baby until the diagnosis is confirmed can increase anxiety in fathers. These factors may contribute to the negative impact of delayed diagnosis fathers’ life satisfaction. 

 The current study has several limitations:

1. The type of CP, feeding method (oral/enteral/parenteral), use of assistive devices, and vision-hearing problems were not assessed in the children with CP included in the study, which may have influenced the findings;
2. When evaluating the findings of this study, it should be kept in mind that family structure and parenting responsibilities may show cultural differences; and
3. The study did not evaluate the extent of fathers’ involvement in the daily care of their children with CP.

The level of direct care provided by fathers to their children with CP may affect fathers’ life satisfaction. 

 In conclusion, this study found that GMFCS level, NSC, and TDC were significant and independent determinants of life satisfaction levels of fathers of children with CP. These findings demonstrate a need for future interventions and research to focus on increasing fathers’ life satisfaction. 

## Data Availability

The database that originated the article is available with the corresponding author.
